# Microscopy Image Browser: A Platform for Segmentation and Analysis of Multidimensional Datasets

**DOI:** 10.1371/journal.pbio.1002340

**Published:** 2016-01-04

**Authors:** Ilya Belevich, Merja Joensuu, Darshan Kumar, Helena Vihinen, Eija Jokitalo

**Affiliations:** Electron Microscopy Unit, Institute of Biotechnology, University of Helsinki, Helsinki, Finland

## Abstract

Understanding the structure–function relationship of cells and organelles in their natural context requires multidimensional imaging. As techniques for multimodal 3-D imaging have become more accessible, effective processing, visualization, and analysis of large datasets are posing a bottleneck for the workflow. Here, we present a new software package for high-performance segmentation and image processing of multidimensional datasets that improves and facilitates the full utilization and quantitative analysis of acquired data, which is freely available from a dedicated website. The open-source environment enables modification and insertion of new plug-ins to customize the program for specific needs. We provide practical examples of program features used for processing, segmentation and analysis of light and electron microscopy datasets, and detailed tutorials to enable users to rapidly and thoroughly learn how to use the program.

## Abstract 2

This Community Page describes a freely available, open-source software that implements and integrates a range of manual and semi-automated segmentation tools for processing and quantifying light and electron microscopy data.

## Introduction

Imaging and image analysis are among the key methods in biosciences nowadays. The knowledge of complex 3-D structures of cells and cell organelles in their natural context is important for understanding the structure–function relationship. Moreover, statistical quantification of 3-D objects based on 2-D image information cannot be reliably made; therefore, segmentation, analysis, visualization, and comparison of whole 3-D volumetric datasets are required. Recently evolved 3-D/5-D light microscopy (LM) and electron microscopy (EM) techniques have enabled a new insight into the morphology of tissues, cells, and cell organelles that had not been conceivable before [1,2]. As the amount of collected data is exponentially increasing, the effectiveness of processing raw data into analyzed results has key importance. There are a number of both commercial (e.g., Amira and Imaris) and freeware image processing packages (e.g., ImageJ [3], Fiji [4], BioImageXD [5], IMOD [6], Ilastik [7], and 3D Slicer [8]) available. However, the performance and usability of image segmentation tools in most packages are still suboptimal, resulting in laborious and time-consuming workflows. Furthermore, with the wide range of software and applications used for data collection and image analysis, there is a clear need for open-access programs that can be adjusted according to the needs of a specific project as well as for cross communication between existing programs.

Here, we present a new open-source software, Microscopy Image Browser (MIB) [9], that was designed for, but not limited to, easy and effective segmentation of multidimensional datasets, improving and facilitating the full utilization and quantitation of acquired data. MIB has a user-friendly graphical interface and is available for all common computer operating systems, either together with MATLAB (Windows, Linux, and Mac OS) or as a stand-alone package for Windows and Mac OS. At present, MIB has been utilized in more than ten different scientific projects, ranging from studies at the cellular level to those dealing with whole organisms; examples include projects on the endoplasmic reticulum (ER) and cytoskeletal filaments in cultured cells [10,11], the organ of Corti in mouse inner ear [12,13], the development of the sieve element in *Arabidopsis thaliana* root [14,15], and the characterization of cryptomonad *Rhinomonas nottbecki* n. sp. [16]. Although MIB was originally designed for the processing of relatively large EM datasets, it can be used for analysis of LM and any other microscopy datasets. Here, we provide several examples highlighting the various features of the program, and online tutorials have been made to provide detailed instructions on how to use them [17].

## MIB Recognizes a Large Number of Imaging Formats and Offers a Variety of Image Processing Tools

**Fig 1:**
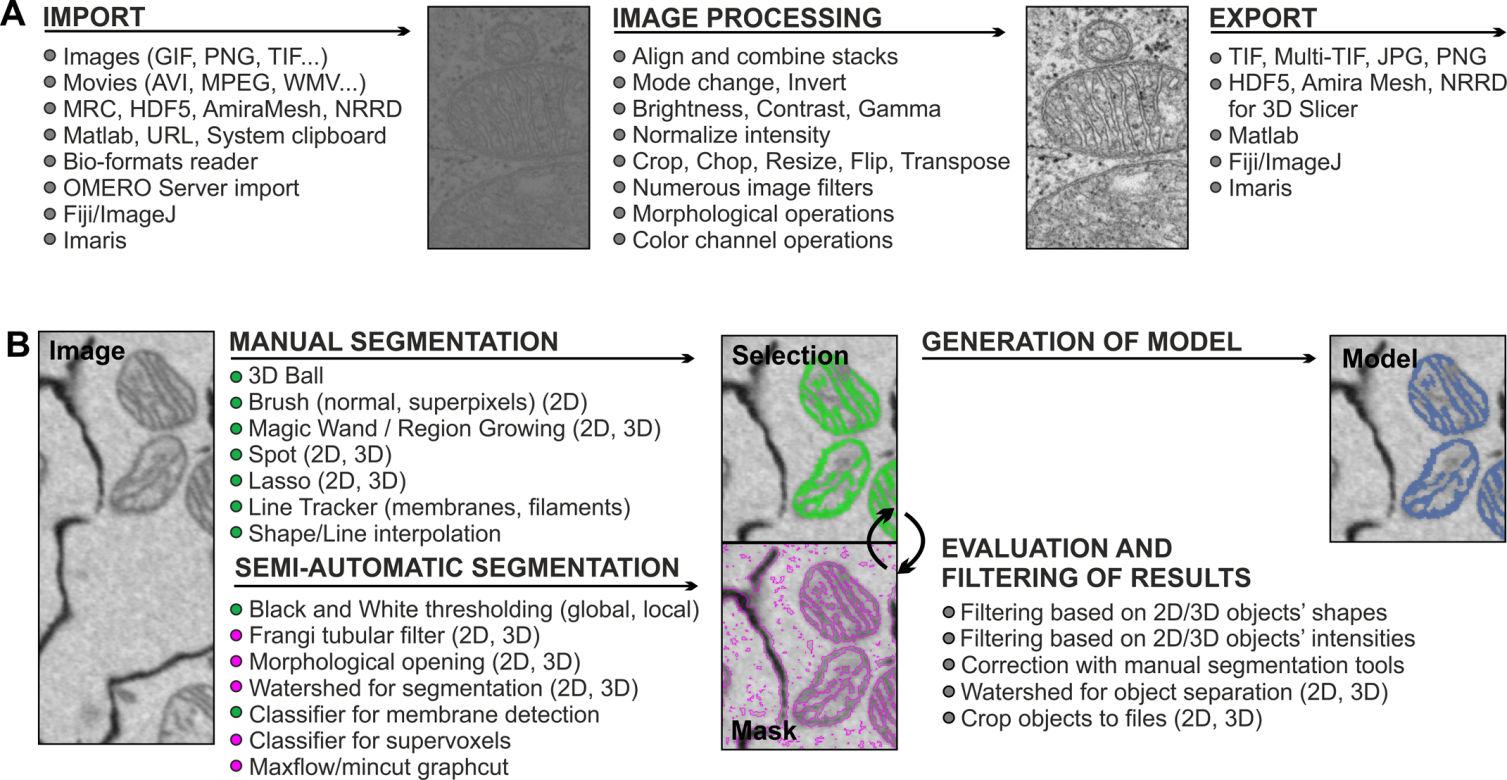
MIB recognizes a large array of imaging formats and offers many essential image processing tools. (A) Most common image formats can be imported to (left column) and exported from (right column) MIB. Most commonly used image processing tools are listed in the middle column (see MIB website for the full list). (B) Image segmentation workflow usually comprises a combination of several approaches. Datasets can be segmented iteratively using various manual and semiautomatic tools combined with quantification filtering of results in order to generate a model. Data in MIB are organized in four layers: Image (raw data), Selection (active layer for segmentation), Mask (an optional supporting layer for temporal storage of the segmentation results for evaluation and filtering), and Model (containing the final segmentation).

The output files from different microscopes and programs are routinely stored in proprietary formats, and access to the collected images and corresponding metadata after acquisition often requires customized software from the manufacturer. MIB overcomes this problem by offering reading capabilities of up to 100 microscopy image and video formats powered by custom-made MATLAB and Bio-Formats [18] readers (Fig 1A; see MIB home page for the full list [9]). MIB was designed as an image browser to allow fast access to individual image datasets for viewing and assembling into 3-D and 4-D stacks (X:Y:Color:Z or X:Y:Color:Time). Up to eight datasets can be simultaneously opened and synchronized, facilitating the comparison and analysis of data from different experiments. The processed images can be exported using most frequently used output formats (Fig 1A).

Quantitative analysis and efficient segmentation of large or otherwise challenging datasets can be facilitated by preprocessing of images (Fig 1A): MIB can resize, flip, rotate, transpose, or crop the datasets as well as adjust brightness, contrast, and gamma settings. In all cases, the voxel (volume pixel) dimensions and the bounding box (exact coordinates of a data block in 3-D space) of the dataset are automatically controlled and adjusted with respect to the performed action. MIB also offers more advanced tools; for example, it has a tool to normalize intensities within the dataset. As intensity levels can easily fluctuate between different images, it is essential to normalize intensities prior to further processing. In many cases, normalization is done based on analysis of intensities of complete slices, while MIB offers additional options for normalization based on preselected areas, which allows normalization even for nonuniform images. Prefiltering of images, especially using anisotropic diffusion filters [19,20], facilitates the segmentation process because of simplification and noise reduction of the images (see MIB homepage for full list of available filters [9]). During a modelling workflow, the filters can be applied to certain arbitrarily shaped (rectangular, ellipsoidal, or polygonal) regions of interest. The alignment feature in MIB can be used to stitch together individual 3-D/4-D datasets while preserving and adjusting the voxel size and bounding box coordinates, correspondingly.

## Multiple Manual and Semiautomatic Tools Are Gathered Together to Enable Segmentation of Various Specimens and Objects of Interest

Biological specimens vary greatly in regard to their shape, size, distribution, and intensity, thereby requiring multiple image segmentation procedures. The image segmentation in MIB combines manual and automatic approaches that may be further polished by filtering the segmented objects using quantification analysis (Fig 1B). This analysis can be easily performed on the shape or intensity properties of the resulting 2-D and 3-D objects. The main manual segmentation tools comprise of brush (normal or against superpixels [21] for faster selection), membrane click tracker (for tracing membrane profiles), magic wand and region growing, spot, and 3-D ball (S1 Video). When using these tools, the researcher interactively follows the modelling process slice by slice. To speed up the manual segmentation, MIB has shape and line interpolation algorithms that fill the gaps between objects drawn on two separated slices. The program also has several semiautomated routines, such as black and white thresholding, that can be applied to the entire dataset or to local selected (masked) area (S1 Video). Selection of such local areas is simple and usually done with the brush tool and the shape interpolation. This approach is especially useful for 3-D EM datasets, as the contrast differences are small and global thresholding (even with quantification filtering) is usually ineffective. The Frangi tubular filter [22] allows the detection of elongated, tubular structures in both 2-D and 3-D space, which is often useful for the segmentation of membranes or vessels.

**Fig 2:**
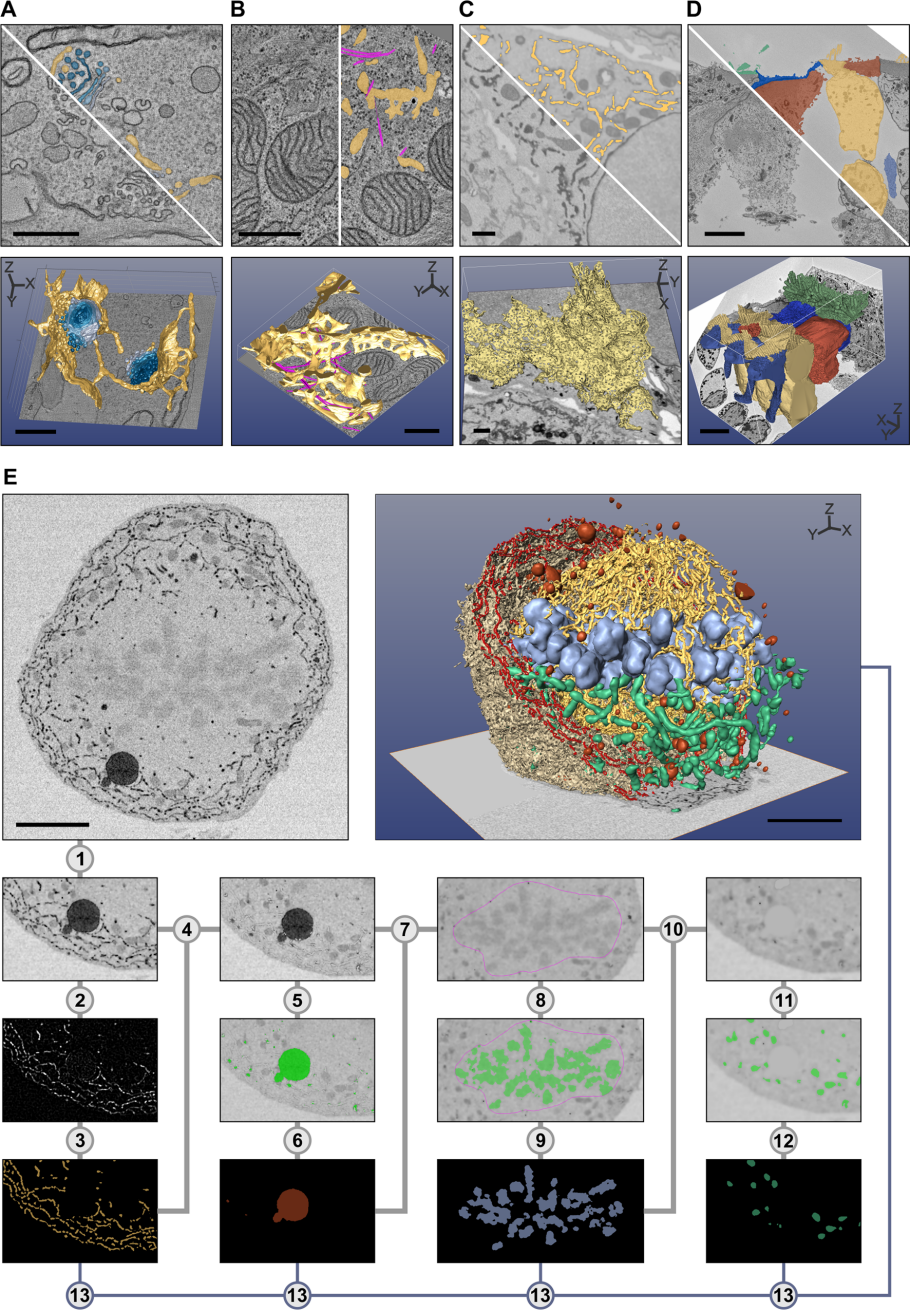
The selection of the best suitable tool for segmentation depends on specimen and object of interest. Five examples are given: in (A–D), the top row of each image shows a preprocessed slice and a segmentation overlay, and the bottom row shows the final 3-D visualization. Segmentation was done with MIB, and the 3-D rendering using different freeware (A–D) and commercial (E) software packages. (A) *Trypanosoma brucei* was chemically fixed and imaged with electron tomography (ET). Each Golgi cisternae (four shades of blue) was manually segmented using the brush tool, while ER and ER-derived vesicles (yellow) were segmented using a combination of the brush tool and shape interpolation. The resulting 3-D model was rendered directly in MIB. (B) A Huh-7 cell was high-pressure frozen and freeze-substituted, and a portion of the cell was subjected to ET [11]. ER (yellow) was segmented using the brush tool with shape interpolation and microtubules (magenta) using the line tracker tool. The resulting model was exported in the IMOD-compatible format and rendered in IMOD [6]. (C) A Huh-7 cell transiently expressing ssHRP-KDEL was cytochemically stained (dark precipitate) and imaged with a serial block-face scanning electron microscope (SB-EM) [11]. The ER network (yellow) was segmented semiautomatically using global black-and-white thresholding and further polished using quantification filtering [11]. The model was exported in the nearly raw raster data (NRRD) format and rendered in 3D Slicer [8]. (D) Mouse cochlea was perilymphatically fixed, and the sensory epithelium of the medial part of the cochlear duct was imaged with SB-EM [13]. Different cell types of the organ of Corti (inner hairs in green, outer hair cells in yellow, external rod in vermilion, internal rod in sapphire blue, and phalangeal part of the Deiters’ cells in greyish blue) were segmented using local thresholding combined with shape interpolation and model rendered using 3D Slicer. (E) Stepwise segmentation workflow is needed to generate a 3-D model of a complex structure. A metaphase Huh-7 cell transiently expressing ssHRP-KDEL was cytochemically stained (dark precipitate in ER lumen) and imaged with SB-EM [10]. The modelling workflow for ER consists of 13 steps (S1 Table). For the visualization, the model was exported in the AmiraMesh format and rendered in Amira. Scale bars: A, B 500 nm; C, D, E 5 μm.

The best suitable segmentation tool depends on the specimen, the imaging method, and the object of interest, and has to be found empirically. As an example, the brush tool was used for segmentation of individual Golgi cisternae (Fig 2A) and, in combination with shape interpolation, for segmentation of vesicles and ER (Fig 2A and 2B), while the line tracker tool was best suited for segmentation of microtubules (Fig 2B). Global thresholding and quantification filtering allow segmentation of high-contrast objects such as cytochemically stained ER (Fig 2C), whereas local thresholding combined with shape interpolation is better suited for segmentation of low-contrast objects such as different cell types in a tissue (Fig 2D). Use of semiautomated tools is essential for segmentation of large datasets and saves time, but as they are not applicable to all datasets, manual methods may be needed for segmentation of images with low-contrast variation, tightly packed areas, or heavily interconnected objects. Typically, modelling of a single dataset requires utilization of multiple segmentation methods. As an example, segmentation of different organelles in a densely packed mitotic cell, which has a high number of objects and is low contrast, is demonstrated by using basic MIB tools (Fig 2E; S1 Table).

## Segmentation of Large Datasets Is Challenging and Requires a Special Set of Tools

Recent advances in both LM and EM have made it possible to acquire large volumetric datasets in a relatively easy manner. Those datasets may vary in size from moderately large volumes of up to 1,000 x 1,000 x 1,000 voxels to huge datasets of about three orders of magnitude higher (e.g., [23,24]). Manual segmentation of such datasets would be tedious, slow, and inefficient, and, as a result, a significant amount of high-quality data may stay unprocessed. Latest developments in image segmentation aim to minimize manual work by implementing automatic approaches of machine learning [25]. A promising example of the machine learning methods for image segmentation is the use of classifiers [7,23,26,27]. Classification starts by manual labelling of representative areas belonging to objects of interest and background. Next, by using various quantitative features (e.g., intensities, texture, and morphology) of the labelled area, the classifier can extend the local segmentation to cover the whole dataset. In some cases, the results can be improved when classification is combined with other methods, for example, the use of supervoxels with watershed [28] or graph-cut-based algorithms [29]. Usually, the use of supervoxels facilitates processing without significant degradation of segmentation results.

**Fig 3:**
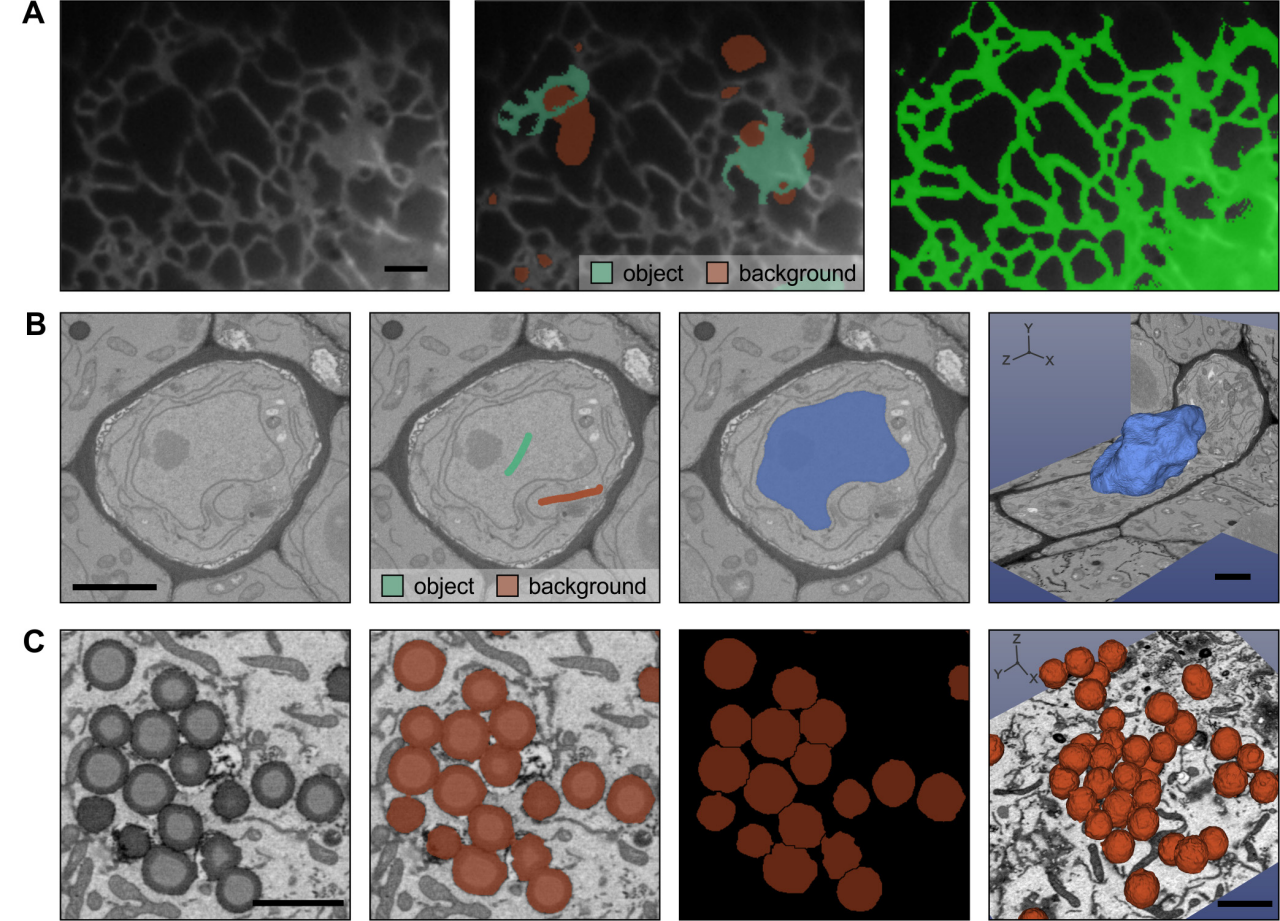
Semiautomatic image segmentation in MIB can dramatically decrease the time spent on modelling. (A) The Random Forest classifier was used to segment ER from wide-field time-lapse LM videos of Huh-7 cells. Labels were assigned (central image) to mark ER (Hsp47-GFP marker seen in green) and background (red), which were then used to train the classifier and segment ER throughout the time-lapse video (right image). (B) The semiautomatic watershed segmentation was used for segmentation of nucleus in the sieve element of *A*. *thaliana* root imaged with SB-EM [14]. Assigning of just two labels (green for nucleus and vermilion for background) was sufficient to segment the complete nucleus in 3-D (light blue, image on the right). (C) The separation of the fused objects using the watershed segmentation. The human U251MG astrocytoma cells were loaded with oleic acid producing a large amount of lipid droplets (LDs) (left image) that tend to form clusters. LDs were segmented using marker-controlled watershed; however, because of close proximity, most of the LDs appear merged (the second image). The object separation mode of the watershed tool was used to separate individual LDs for quantitative analysis (third and fourth images). The 3-D models were rendered with 3D Slicer [8]. Scale bars: 2 μm.

MIB currently includes two classifiers. The first one (called membrane detection) is based on a protocol developed by Kaynig et al. [27] for membrane detection from EM images. However, it is also suitable for segmentation of organelles from EM (S2 Video) and LM datasets (Fig 3A). We have used it to detect ER from time-lapse LM videos when, because of the gradient of the background intensities and variety of shapes (sheets and tubules), the standard thresholding methods are insufficient. The second classifier (called supervoxel classification) was designed to be fast and is based on classification of superpixels (2-D images) or supervoxels (3-D volumes) generated by the simple linear iterative clustering (SLIC) algorithm [21]. This classification uses only basic image intensity properties (e.g., minimal, maximal, average intensity, and cuts through the histogram) of each supervoxel and its neighbours. It is powerful in detection of objects that have distinct intensity features, for example, fluorescently labelled cells or organelles.

A graph-based semiautomatic segmentation in MIB starts by clustering of voxels to supervoxels [21] that are assigned to vertices of a graph, where the edges connecting vertices are defined by the difference between average intensities of the corresponding supervoxels. Once the user interactively labels the areas belonging to the target and background, MIB uses the maxflox/min-cut algorithm [30] to perform the segmentation. For objects that have distinct boundaries, the watershed transformation [31] is extremely efficient. The marker-controlled watershed segmentation can be applied to detect both 2-D and 3-D objects. In practice, labelling the object(s) and background on a single slice allows segmentation of membrane-enclosed organelles in 3-D, as demonstrated for segmentation of the nucleus (Fig 3B and S2 Video) [14]. Objects that do not have clear boundaries and therefore would not qualify for segmentation using watershed can be preprocessed with the gradient filter to generate the required boundaries. Watershed can also be used to separate merged 2-D and 3-D objects even in anisotropic datasets [32], as exemplified by the separation of lipid droplets (LDs) (Fig 3C). For processing of large volumes, the chop tool can be used to divide the initial volume into smaller datasets for parallel segmentation on multiple workstations. Import of the chopped images then automatically assembles the segmented blocks together.

In automated segmentation, an essential part of the workflow is the estimation of accuracy of the applied method. As MIB was designed for segmentation of various objects coming from multiple imaging modalities, finding suitable parameters for accuracy metrics would be challenging, and the current version does not offer any. Therefore, MIB is most suitable for the processing of moderately large datasets, where the workflow can still be interactive and a researcher approves each step iteratively.

## 3-D Visualization and Quantification of Images and Models Are the Final Steps in Imaging

**Fig 4:**
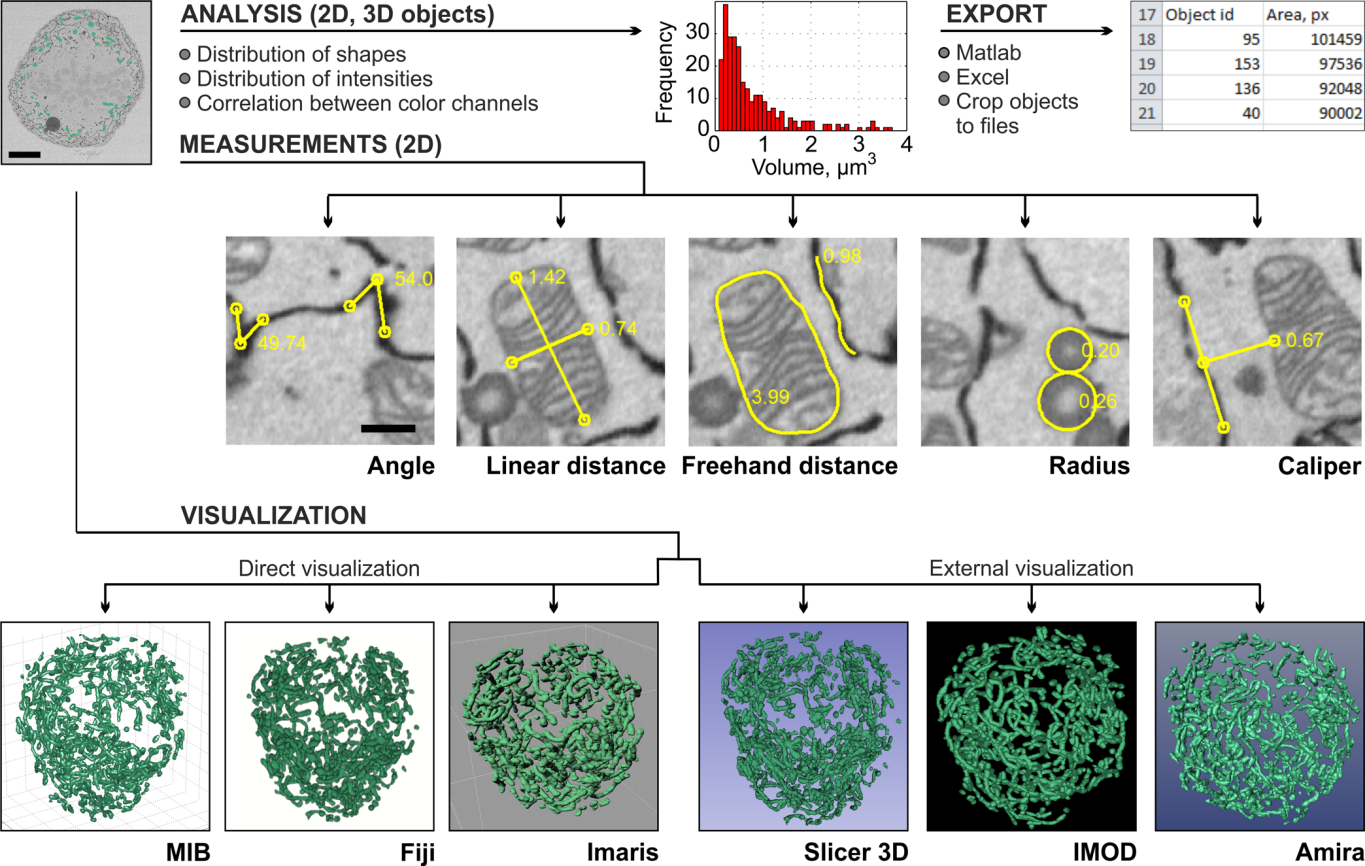
Quantification and visualization of results are the final steps of the imaging workflow. Generated models may be quantified to extract different parameters of the segmented objects and visualized using a number of programs. As an example, the volumes (μm^3^) and numbers of segmented mitochondria were calculated (the plot in red). The quantifications results can either be plotted directly in MIB or exported to MATLAB or Microsoft Excel. The manual measurements of angles, distances, caliper, and radius complement automatic quantification. The visualization of the mitochondria model (in green) is demonstrated using six alternative programs (the lower row).

3-D visualization is an important part of any modelling. In contrast to segmentation, there are good 3-D visualization programs available already. While MIB can be used to visualize the models (Fig 2A), programs specialized to visualization might be more suitable. From MIB, the results of the segmentation (models) can be directly visualized using MATLAB engine (Fig 2A), Fiji 3-D viewer [33,34], and Imaris or saved in formats that are compatible with other visualization programs, i.e., IMOD (Fig 2B) [6,35], Amira (Fig 2E), or 3D Slicer (Fig 2C and 2D; Fig 3B and 3C) [8]. In other cases, the models may be saved in the TIF format for free packages such as BioImageXD [5], Drishti [36], or Vaa3D (Fig 4) [37].

For full utilization of the acquired data, the visualization should be complemented with numerical analysis. MIB provides several tools for numerical analysis of shapes (e.g., size, eccentricity, perimeter, and orientation) and intensities (e.g., minimum, maximum, and average) of 2-D and 3-D objects. These calculated parameters can either be used for filtering of objects as a part of the segmentation protocol or be exported to MATLAB and Microsoft Excel (Fig 4) for further statistical analysis. When needed, the automatic measurements can be complemented with the manual measurements of angles, distances, and radii (Fig 4) that also include an intensity profile of the image under the measured areas.

## MIB Is a Freely Distributed Open-Source Program with a User-Friendly Graphical User Interface

MIB is written with MATLAB, which is available for all common computer operating systems (Windows, Linux, and Mac OS). As a high-level scientific programming language, MATLAB’s program code is easy to understand, and, thus, its use for implementing image-processing routines is fast and cost efficient, not only for initial development but also for any enhancements of MIB with additional features, which can be achieved because of MIB’s open-source code. MATLAB has a large community of users who are developing and sharing algorithms using MATLAB Central File Exchange [38], an online library of over 20,000 user-contributed MATLAB files and toolboxes. Many of these submissions are dedicated to image processing and may easily be integrated into MIB, for example, as plug-ins. To support the development of plug-ins, MIB includes a description of its application programming interface that includes tutorials. MIB automatically detects all available plug-ins and initializes them during program start-up. For those researchers who are not familiar with MATLAB or who do not have a MATLAB license, we provide MIB as a stand-alone package (64-bit Windows and Mac OS) that can be freely downloaded and used on standard computers.

**Fig 5:**
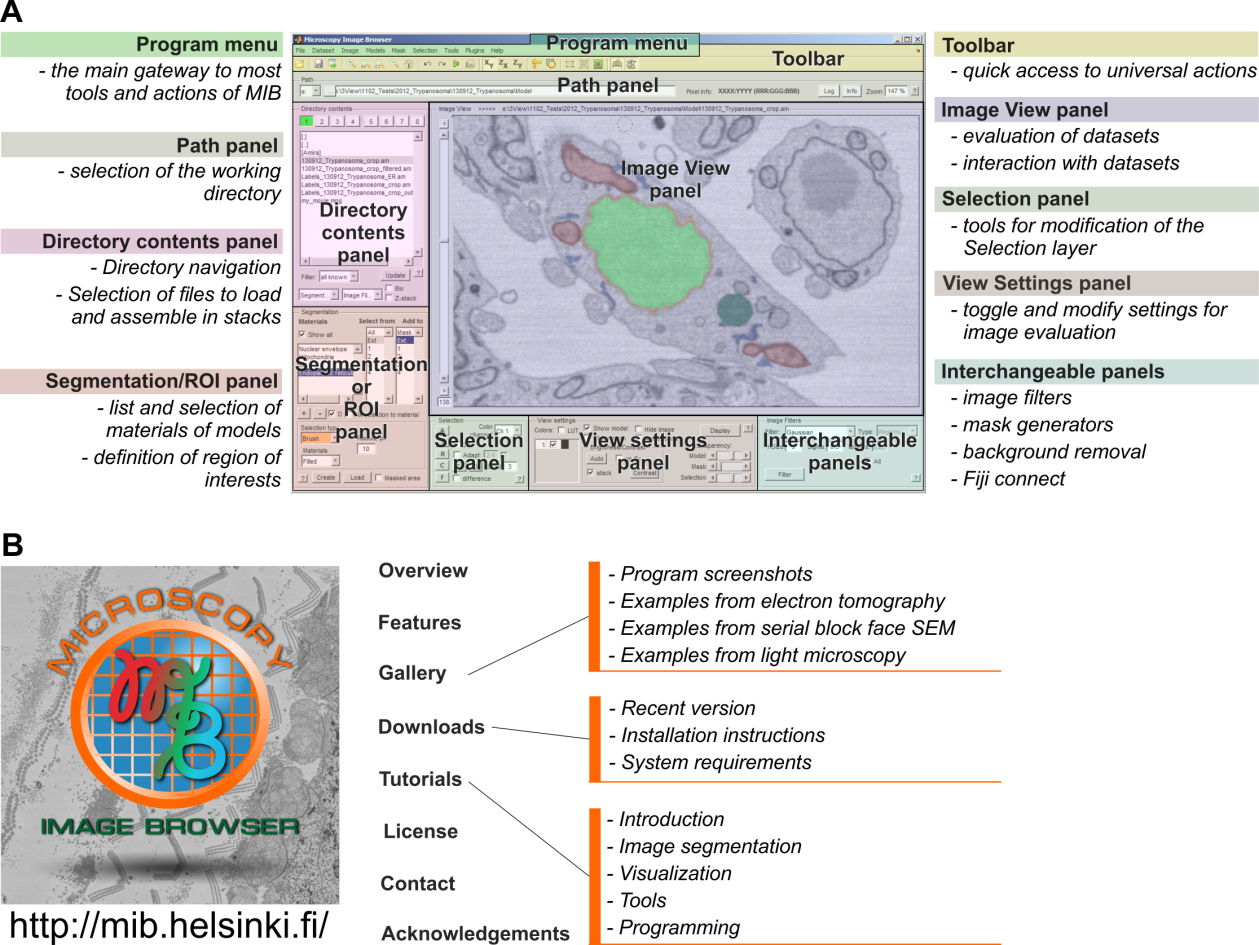
MIB has a user-friendly graphical user interface and is freely available from the website. (A) A screenshot of the MIB user interface. The program menu, toolbar, and panels are highlighted. A brief description of each element is provided. (B) A dedicated website includes direct links for software download and covers various topics and aspects of MIB functionality. *Image credit*: *Ilya Belevich*, *on behalf of MIB*.

MATLAB itself is mostly a collection of functions that may be difficult to use by novices. To improve the usability, MIB includes an intuitive graphical user interface with standard components such as a menu, toolbar, and panels in the main window (Fig 5A). The toolbar and panels provide fast access to the most essential features described earlier, while the menu is used to access less frequently used actions. Some of the panels can be changed to adapt to the specific needs of a scientific project. MIB is designed to take the challenges of color-blind researchers into account, as we have selected a default color palette in which each color appears as a distinctive shade for color-blind users [39]. Alternatively, the default color palette can easily be replaced by one of the other color-blind-friendly ready assembled palettes [40] or each color can be individually selected. Each image-processing manipulation is logged, and the logs are stored with the data for future reference. To overcome accidental errors or to test most suitable tools and parameters during image processing, MIB has a flexible undo system that can erase recent changes made to the data or model. The number of undo steps for both 2-D and 3-D actions can be individually customized, thereby allowing optimized memory usage. MIB has a large variety of key shortcuts that improve segmentation performance and minimize unnecessary mouse movements. All details about the program usage are well documented and available from online tutorials on the MIB website (Fig 5B) [17] or from a built-in help system accessed through the Help menu or via dedicated buttons in each panel and auxiliary windows.

One of our aims during MIB development was to create a freely distributed open-source platform that would also allow a smooth cross talk with other image-processing programs. Depending on the program, the integration was accomplished with either direct data exchange or by data format compatibilities. The open microscopy environment (OMERO) image server [41] can be directly accessed to download data, whereas the direct data exchange [33] with Imaris and Fiji [4] allows a large collection of their plug-ins to be used without generating space-consuming intermediate files. In cases where the direct link is not possible, the compatibility is achieved through use of common file formats. We anticipate that MIB will become a useful tool not only for biological researchers but also for mathematicians as a platform for implementation of new methods for image segmentation developed in MATLAB. We will also keep on upgrading and extending MIB with each new imaging project.

## Supporting Information

S1 Methods A description of cell lines, constructs, specimen preparation, and imaging conditions. (DOCX)

S1 Table Segmentation of a densely packed mitotic cell requires utilization of various segmentation tools provided by MIB (Fig 2E). First, the data was filtered using anisotropic diffusion filter (adapted from http://www.peterkovesi.com/matlabfns/) to eliminate the local noise while preserving the edges of the objects (step 1). As the ER network is extensive and makes contacts with nearly all other organelles, the removal of ER facilitates segmentation of all the other organelles later. Also in this example, the ER has a high contrast because of the luminal cytochemical staining making its segmentation feasible. In step 2, the morphological bottom-hat filter was used to temporally remove all large objects (such as mitochondria, chromosomes, and LDs). The Frangi vessel enhancement filter (adapted from http://www.mathworks.com/matlabcentral/fileexchange/24409-hessian-based-frangi-vesselness-filter) was then applied sequentially in the XY and ZX planes to segment ER tubules and sheet remnants (step 3: generation of the ER model). Next, the areas belonging to the ER in the anisotropically filtered image (from step 1) were replaced by the background color to generate degraded image without the ER (step 4). The resulting image was thresholded to select the dark singular objects such as LDs, peroxisomes, and lysosomes (step 5). To eliminate small objects, e.g., vesicles, the segmented areas were further smoothed using erosion followed by dilation in 3-D (step 6: generation of models for lysosomes, peroxisomes, and LDs). These areas were then replaced by the background color, similarly as in step 4 (step 7). As equatorially aligned chromosomes have contrast quite close to the mitochondria, global thresholding could not be used to discriminate them. Therefore, by using the brush tool and the shape interpolation, the central area of the cell was masked and thresholded to segment the chromosomes (step 8). The chromosomes were smoothed similarly as in step 6 (step 9: generation of the chromosome model). The chromosomal areas were replaced by the background color, and additional anisotropic diffusion filtering was applied (step 10). Segmentation of mitochondria was done by using the morphological image opening and thresholding (step 11), followed by smoothing and filtration of small objects (step 12: generation of the mitochondria model). The final model was assembled by combining all individual models and then visualized (step 13).
(DOCX)

S2 Table List of all third-party tools and functions, including URL links. (DOCX)

S1 Video Basic segmentation tools of MIB. The video demonstrates the use of different basic image segmentation tools: (A) 3-D ball: modelling of LDs (00:04), (B) brush: modelling of Golgi stack (00:37), (C) brush with superpixels: segmentation of cells from LM (01:21), (C) magic wand: modelling of ER (01:50), (E) line tracker in 3-D: modelling of microtubules (02:22), (F) line tracker in 2-D and line interpolation: modelling of nuclear envelope (03:13), (G) shape interpolation: modelling of mitochondria (04:04), and (H) global and local black and white thresholding: modelling of nuclear envelope with nuclear pores (04:38). Each clip contains screen capture taken during the segmentation process and the final 3-D visualization of the model. The starting point of each clip is given in brackets.
(MP4)

S2 Video Advanced segmentation tools of MIB. The video demonstrates the use of advanced image segmentation tools: (A) random forest classifier: modelling of ER (00:01) and (B) watershed: modelling of a nucleus (01:23). Each clip contains screen captures taken during the segmentation process and the final 3-D visualization of the model. The starting point of each clip is given in brackets.
(MP4)

## Abbreviations

EM: electron microscopy
ET: electron tomography
LD: lipid droplet
LM: light microscopy
MIB: Microscopy Image Browser
NRRD: nearly raw raster data
OMERO: open microscopy environment
SB-EM: serial block-face scanning electron microscope
SLIC: simple linear iterative clustering

## References

[1] Knott G, Genoud C. Is EM dead? Journal of cell science. 2013;126(Pt 20):4545–52. 10.1242/jcs.124123 . 24124192

[2] Lidke DS, Lidke KA. Advances in high-resolution imaging—techniques for three-dimensional imaging of cellular structures. Journal of cell science. 2012;125(Pt 11):2571–80. 10.1242/jcs.090027 22685332 PMC3706075

[3] Schneider CA, Rasband WS, Eliceiri KW. NIH Image to ImageJ: 25 years of image analysis. Nat Methods. 2012;9(7):671–5. 10.1038/nmeth.2089 . 22930834 PMC5554542

[4] Schindelin J, Arganda-Carreras I, Frise E, Kaynig V, Longair M, Pietzsch T, et al Fiji: an open-source platform for biological-image analysis. Nat Methods. 2012;9(7):676–82. 10.1038/Nmeth.2019 . 22743772 PMC3855844

[5] Kankaanpää P, Paavolainen L, Tiitta S, Karjalainen M, Päivärinne J, Nieminen J, et al BioImageXD: an open, general-purpose and high-throughput image-processing platform. Nat Methods. 2012;9(7):683–9. 10.1038/nmeth.2047 . 22743773

[6] Kremer JR, Mastronarde DN, McIntosh JR. Computer visualization of three-dimensional image data using IMOD. Journal of structural biology. 1996;116(1):71–6. 10.1006/jsbi.1996.0013 . 8742726

[7] Sommer C, Straehle C, Kothe U, Hamprecht FA. Ilastik: Interactive Learning and Segmentation Toolkit. I S Biomed Imaging. 2011:230–3. .

[8] Fedorov A, Beichel R, Kalpathy-Cramer J, Finet J, Fillion-Robin JC, Pujol S, et al 3D Slicer as an image computing platform for the Quantitative Imaging Network. Magnetic resonance imaging. 2012;30(9):1323–41. 10.1016/j.mri.2012.05.001 22770690 PMC3466397

[9] Belevich I, Joensuu M, Kumar D, Vihinen H, Jokitalo E. Microscopy Image Browser 2015. http://mib.helsinki.fi.

[10] Puhka M, Joensuu M, Vihinen H, Belevich I, Jokitalo E. Progressive sheet-to-tubule transformation is a general mechanism for endoplasmic reticulum partitioning in dividing mammalian cells. Molecular biology of the cell. 2012;23(13):2424–32. 10.1091/mbc.E10-12-0950 22573885 PMC3386207

[11] Joensuu M, Belevich I, Rämö O, Nevzorov I, Vihinen H, Puhka M, et al ER sheet persistence is coupled to myosin 1c-regulated dynamic actin filament arrays. Molecular biology of the cell. 2014;25(7):1111–26. 10.1091/mbc.E13-12-0712 24523293 PMC3967974

[12] Anttonen T, Belevich I, Kirjavainen A, Laos M, Brakebusch C, Jokitalo E, et al How to bury the dead: elimination of apoptotic hair cells from the hearing organ of the mouse. Journal of the Association for Research in Otolaryngology: JARO. 2014;15(6):975–92. 10.1007/s10162-014-0480-x 25074370 PMC4389953

[13] Anttonen T, Kirjavainen A, Belevich I, Laos M, Richardson WD, Jokitalo E, et al Cdc42-dependent structural development of auditory supporting cells is required for wound healing at adulthood. Scientific reports. 2012;2:978 10.1038/srep00978 23248743 PMC3523287

[14] Furuta KM, Yadav SR, Lehesranta S, Belevich I, Miyashima S, Heo JO, et al Plant development. Arabidopsis NAC45/86 direct sieve element morphogenesis culminating in enucleation. Science. 2014;345(6199):933–7. 10.1126/science.1253736 . 25081480

[15] Dettmer J, Ursache R, Campilho A, Miyashima S, Belevich I, O'Regan S, et al CHOLINE TRANSPORTER-LIKE1 is required for sieve plate development to mediate long-distance cell-to-cell communication. Nature communications. 2014;5:4276 10.1038/ncomms5276 . 25008948

[16] Majaneva M, Remonen I, Rintala JM, Belevich I, Kremp A, Setälä O, et al Rhinomonas nottbecki n. sp. (Cryptomonadales) and Molecular Phylogeny of the Family Pyrenomonadaceae. The Journal of eukaryotic microbiology. 2014;61(5):480–92. 10.1111/jeu.12128 . 24913840

[17] Belevich I, Kumar D, Vihinen H. Microscopy Image Browser, on-line tutorials 2015. http://mib.helsinki.fi/tutorials.html.

[18] Linkert M, Rueden CT, Allan C, Burel JM, Moore W, Patterson A, et al Metadata matters: access to image data in the real world. The Journal of cell biology. 2010;189(5):777–82. 10.1083/jcb.201004104 20513764 PMC2878938

[19] Perona P, Malik J. Scale-Space and Edge-Detection Using Anisotropic Diffusion. Ieee T Pattern Anal. 1990;12(7):629–39. 10.1109/34.56205 .

[20] Kroon DJ, Slump CH, Maal TJJ. Optimized Anisotropic Rotational Invariant Diffusion Scheme on Cone-Beam CT. Medical Image Computing and Computer-Assisted Intervention—Miccai 2010, Pt Iii. 2010;6363:221–8. 10.1007/978-3-642-15711-0_28 . 20879403

[21] Achanta R, Shaji A, Smith K, Lucchi A, Fua P, Susstrunk S. SLIC superpixels compared to state-of-the-art superpixel methods. IEEE Trans Pattern Anal Mach Intell. 2012;34(11):2274–82. 10.1109/TPAMI.2012.120 . 22641706

[22] Frangi AF, Niessen WJ, Vincken KL, Viergever MA. Multiscale vessel enhancement filtering. Medical Image Computing and Computer-Assisted Intervention—Miccai'98. 1998;1496:130–7. .

[23] Perez AJ, Seyedhosseini M, Deerinck TJ, Bushong EA, Panda S, Tasdizen T, et al A workflow for the automatic segmentation of organelles in electron microscopy image stacks. Frontiers in neuroanatomy. 2014;8:126 10.3389/fnana.2014.00126 25426032 PMC4224098

[24] Helmstaedter M, Briggman KL, Turaga SC, Jain V, Seung HS, Denk W. Connectomic reconstruction of the inner plexiform layer in the mouse retina. Nature. 2013;500(7461):168–74. 10.1038/nature12346 . 23925239

[25] Sommer C, Gerlich DW. Machine learning in cell biology—teaching computers to recognize phenotypes. Journal of cell science. 2013;126(Pt 24):5529–39. 10.1242/jcs.123604 . 24259662

[26] Breiman L. Random forests. Mach Learn. 2001;45(1):5–32. 10.1023/A:1010933404324 .

[27] Kaynig V, Fuchs T, Buhmann JM. Neuron Geometry Extraction by Perceptual Grouping in ssTEM Images. Proc Cvpr Ieee. 2010:2902–9. 10.1109/Cvpr.2010.5540029 .

[28] Jones C, Liu T, Cohan NW, Ellisman M, Tasdizen T. Efficient semi-automatic 3-D segmentation for neuron tracing in electron microscopy images. Journal of neuroscience methods. 2015;246:13–21. 10.1016/j.jneumeth.2015.03.005 25769273 PMC4398646

[29] Lucchi A, Smith K, Achanta R, Knott G, Fua P. Supervoxel-based segmentation of mitochondria in em image stacks with learned shape features. IEEE transactions on medical imaging. 2012;31(2):474–86. 10.1109/TMI.2011.2171705 . 21997252

[30] Boykov Y, Kolmogorov V. An experimental comparison of min-cut/max-flow algorithms for energy minimization in vision. IEEE Trans Pattern Anal Mach Intell. 2004;26(9):1124–37. 10.1109/TPAMI.2004.60 . 15742889

[31] Couprie C, Grady L, Najman L, Talbot H. Power Watersheds: A Unifying Graph-Based Optimization Framework. IEEE Trans Pattern Anal Mach Intell. 2010 10.1109/TPAMI.2010.200 . 21079274

[32] Mishchenko Y. A fast algorithm for computation of discrete Euclidean distance transform in three or more dimensions on vector processing architectures. SIViP. 2015;9(1):19–27. 10.1007/s11760-012-0419-9

[33] Sage D, Prodanov D, J.-Y. T, Schindelin J. MIJ: Making Interoperability Between ImageJ and Matlab Possible. ImageJ User & Developer Conference; Luxembourg2012.

[34] Schmid B, Schindelin J, Cardona A, Longair M, Heisenberg M. A high-level 3-D visualization API for Java and ImageJ. Bmc Bioinformatics. 2010;11:274 10.1186/1471-2105-11-274 20492697 PMC2896381

[35] Nicastro D, Schwartz C, Pierson J, Gaudette R, Porter ME, McIntosh JR. The molecular architecture of axonemes revealed by cryoelectron tomography. Science. 2006;313(5789):944–8. 10.1126/science.1128618 . 16917055

[36] Drishti: volume exploration and presentation tool. http://sf.anu.edu.au/Vizlab/drishti.

[37] Peng H, Ruan Z, Long F, Simpson JH, Myers EW. V3-D enables real-time 3-D visualization and quantitative analysis of large-scale biological image data sets. Nature biotechnology. 2010;28(4):348–53. 10.1038/nbt.1612 20231818 PMC2857929

[38] MathWorks. File exchange. http://www.mathworks.se/matlabcentral/fileexchange/.

[39] Ichihara YG, Okabe M, Iga K, Tanaka Y, Musha K, Ito K. Color Universal Design—The selection of four easily distinguishahle colors for all color vision types. Proc Spie. 2008;6807 10.1117/12.765420 .

[40] Brewer C, Harrower M, Sheesley B, Woodruff A, Heyman D. ColorBrewer 2.0 2013. http://colorbrewer2.org/.

[41] Allan C, Burel JM, Moore J, Blackburn C, Linkert M, Loynton S, et al OMERO: flexible, model-driven data management for experimental biology. Nat Methods. 2012;9(3):245–53. 10.1038/nmeth.1896 22373911 PMC3437820

