# Supplementary material for: Microscopy Image Browser: A Platform for Segmentation and Analysis of Multidimensional Datasets
Source: PLoS Biol. 2016 Jan 4;14(1):e1002340. doi: 10.1371/journal.pbio.1002340 (PMC4699692; doi:10.1371/journal.pbio.1002340)
Supplement: S1 Methods — (DOCX) [file pbio.1002340.s001.docx]

**Supplementary materials and Methods**

**Cell cultures, tissues and constructs**

Human hepatoma cells (Huh-7, JCRB0403; Japanese Collection of Research Bioresources Cell Bank, Osaka, Japan) were cultured as previously described [1]. Human U251MG astrocytoma cells were grown in RPMI 1640 medium supplemented with 10% fetal bovine serum, L-glutamine, and 100 IU/ml penicillin and 100 µg/ml streptomycin (all from BioWhittaker, Lonza, Basel, Switzerland. When indicated, cells were transiently expressing Hsp47-GFP-KDEL [2] or ssHRP-KDEL [3]. Cytochemical staining of cells expressing ssHRP-KDEL was described earlier [4]. To induce formation of LD the cells were supplemented for 15 minutes with 200 µM oleic acid as 8:1-molar-ratio BSA complex prepared in serum-free DMEM.

*Trypanosoma brucei* cells were cultured in Cunningham media containing 15% heat-inactivated fetal calf serum (Hyclone) at 28°C. Phloem sieve element cells of *Arabidopsis thaliana* WT Columbia (Col-0) were grown and dissected as described earlier [5]. Cochlea of the inner ear were dissected from NMRI mice at embryonic day 18 or postnatal day 21 as described earlier [6].

**Specimen preparation and imaging**

Live wide-field images of Huh-7 cells were acquired as described previously [7]. 2 or 3 successive 250-nm sections from Huh-7 cells prepared using high-pressure freezing and freeze substitution [7] or from *T. brucei* using chemical fixation and plastic embedding [8], respectively, were subjected to electron tomography as described previously [7]. Dual tilt series, ±62 degrees were acquired at nominal magnification of 9600x and 11500x, and binned twice providing a pixel size of 2.3 nm and 1.9 nm, respectively. SB-EM specimen preparation and imaging conditions for Huh-7 cells [1], *A. thaliana* roots [5], and mouse cochlea [9] have been described earlier.

**Image processing, and visualization programs**

All datasets were fully processed using Microscopy Image Browser. MIB was tested with MATLAB, releases 2011a - 2015b. The supplementary videos were generated using Amira 5.6 and screen capture of the MIB interface and assembled in Sony Movie Studio 11 (http://www.sonycreativesoftware.com/moviestudio). Visualization of 3D models and volumes was done using: **a)** MATLAB direct visualization from MIB with help of view3d written by Torsten Vogel (http://www.mathworks.com/matlabcentral/fileexchange/334-view3d-m); **b)** IMOD 4.7.14 [10]; **c)** Slicer 3D 4.4.0 [11]; **d)** Amira 5.6 (FEI Company); **e)** Fiji 3D viewer 1.49q [12]; **f)** Imaris 8.0.2 (Bitplane, Oxford Instruments)

1. Puhka M, Joensuu M, Vihinen H, Belevich I, Jokitalo E. Progressive sheet-to-tubule transformation is a general mechanism for endoplasmic reticulum partitioning in dividing mammalian cells. Molecular biology of the cell. 2012;23(13):2424-32, doi: 10.1091/mbc.E10-12-0950.

2. Kano F, Kondo H, Yamamoto A, Kaneko Y, Uchiyama K, Hosokawa N, et al. NSF/SNAPs and p97/p47/VCIP135 are sequentially required for cell cycle-dependent reformation of the ER network. Genes to cells : devoted to molecular & cellular mechanisms. 2005;10(10):989-99, doi: 10.1111/j.1365-2443.2005.00894.x.

3. Connolly CN, Futter CE, Gibson A, Hopkins CR, Cutler DF. Transport into and out of the Golgi complex studied by transfecting cells with cDNAs encoding horseradish peroxidase. The Journal of cell biology. 1994;127(3):641-52, doi:

4. Jokitalo E, Cabrera-Poch N, Warren G, Shima DT. Golgi clusters and vesicles mediate mitotic inheritance independently of the endoplasmic reticulum. The Journal of cell biology. 2001;154(2):317-30, doi: 10.1083/jcb.200104073.

5. Dettmer J, Ursache R, Campilho A, Miyashima S, Belevich I, O'Regan S, et al. CHOLINE TRANSPORTER-LIKE1 is required for sieve plate development to mediate long-distance cell-to-cell communication. Nature communications. 2014;5:4276, doi: 10.1038/ncomms5276.

6. Anttonen T, Belevich I, Kirjavainen A, Laos M, Brakebusch C, Jokitalo E, et al. How to bury the dead: elimination of apoptotic hair cells from the hearing organ of the mouse. Journal of the Association for Research in Otolaryngology : JARO. 2014;15(6):975-92, doi: 10.1007/s10162-014-0480-x.

7. Joensuu M, Belevich I, Ramo O, Nevzorov I, Vihinen H, Puhka M, et al. ER sheet persistence is coupled to myosin 1c-regulated dynamic actin filament arrays. Molecular biology of the cell. 2014;25(7):1111-26, doi: 10.1091/mbc.E13-12-0712.

8. Yelinek JT, He CY, Warren G. Ultrastructural study of Golgi duplication in Trypanosoma brucei. Traffic. 2009;10(3):300-6, doi: 10.1111/j.1600-0854.2008.00873.x.

9. Anttonen T, Kirjavainen A, Belevich I, Laos M, Richardson WD, Jokitalo E, et al. Cdc42-dependent structural development of auditory supporting cells is required for wound healing at adulthood. Scientific reports. 2012;2:978, doi: 10.1038/srep00978.

10. Kremer JR, Mastronarde DN, McIntosh JR. Computer visualization of three-dimensional image data using IMOD. Journal of structural biology. 1996;116(1):71-6, doi: 10.1006/jsbi.1996.0013.

11. Fedorov A, Beichel R, Kalpathy-Cramer J, Finet J, Fillion-Robin JC, Pujol S, et al. 3D Slicer as an image computing platform for the Quantitative Imaging Network. Magnetic resonance imaging. 2012;30(9):1323-41, doi: 10.1016/j.mri.2012.05.001.

12. Schmid B, Schindelin J, Cardona A, Longair M, Heisenberg M. A high-level 3D visualization API for Java and ImageJ. Bmc Bioinformatics. 2010;11:274, doi: 10.1186/1471-2105-11-274.
