# Supplementary material for: Microscopy Image Browser: A Platform for Segmentation and Analysis of Multidimensional Datasets
Source: PLoS Biol. 2016 Jan 4;14(1):e1002340. doi: 10.1371/journal.pbio.1002340 (PMC4699692; doi:10.1371/journal.pbio.1002340)
Supplement: S1 Table — First, the data was filtered using anisotropic diffusion filter (adapted from http://www.peterkovesi.com/matlabfns/) to eliminate the local noise while preserving the edges of the objects (step 1). As the ER network is extensive and makes contacts with nearly all other organelles, the removal of ER facilitates segmentation of all the other organelles later. Also in this example, the ER has a high contrast because of the luminal cytochemical staining making its segmentation feasible. In step 2, the morphological bottom-hat filter was used to temporally remove all large objects (such as mitochondria, chromosomes, and LDs). The Frangi vessel enhancement filter (adapted from http://www.mathworks.com/matlabcentral/fileexchange/24409-hessian-based-frangi-vesselness-filter) was then applied sequentially in the XY and ZX planes to segment ER tubules and sheet remnants (step 3: generation of the ER model). Next, the areas belonging to the ER in the anisotropically filtered image (from step 1) were replaced by the background color to generate degraded image without the ER (step 4). The resulting image was thresholded to select the dark singular objects such as LDs, peroxisomes, and lysosomes (step 5). To eliminate small objects, e.g., vesicles, the segmented areas were further smoothed using erosion followed by dilation in 3-D (step 6: generation of models for lysosomes, peroxisomes, and LDs). These areas were then replaced by the background color, similarly as in step 4 (step 7). As equatorially aligned chromosomes have contrast quite close to the mitochondria, global thresholding could not be used to discriminate them. Therefore, by using the brush tool and the shape interpolation, the central area of the cell was masked and thresholded to segment the chromosomes (step 8). The chromosomes were smoothed similarly as in step 6 (step 9: generation of the chromosome model). The chromosomal areas were replaced by the background color, and additional anisotropic diffusion filteri [file pbio.1002340.s002.docx]

| **Step** | **Action** | **Parameters** | **Snapshot** |
| --- | --- | --- | --- |
| 0. | **Load the dataset**  *Where in MIB:*  ***a)*** *Select files in the “Directory Contents Panel” using the left mouse button*  ***b)*** *Press the right mouse button to call a context menu*  ***c)*** *Choose the “Combine selected datasets” option* | NA | 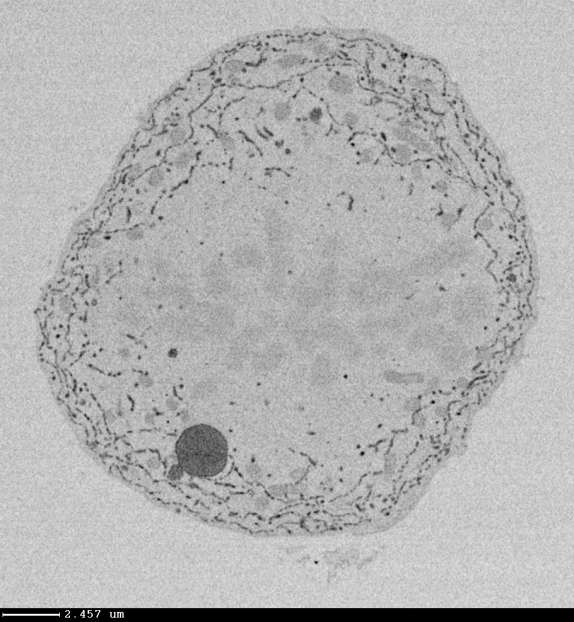 |
| 1. | **Perona-Malik**  **anisotropic diffusion filtering**  to eliminate local noise while preserving edges of organelles  *Where in MIB:*  ***a)*** *Image Filters Panel→Perona Malik anisotropic diffusion→Filter*  ***b)*** *Save the filtered dataset to the hard drive for future use under a different filename, Menu→File→Save As* | Type: Regions  Number of iterations (Iter): 10  Edge stopping parameter (K): 4  Diffusion step (lambda): 0.15  All: on | 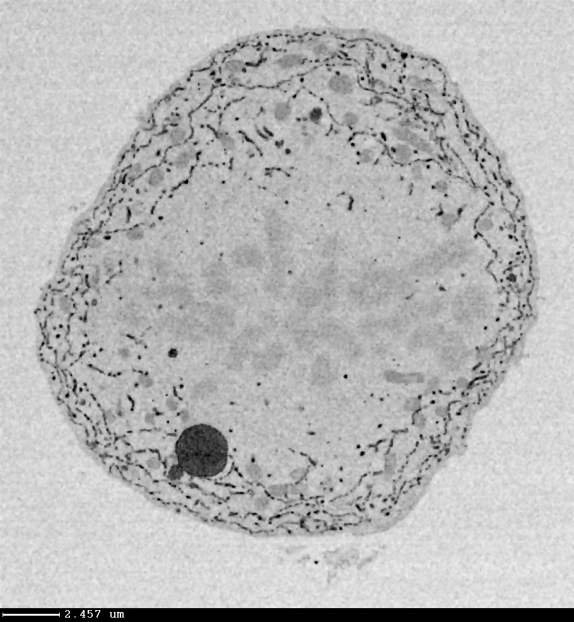 |
| 2. | **Morphological bottom-hat filtering**  to temporally remove all large organelles such as chromosomes and mitochondria, while keeping ER  *Where in MIB:*  *Menu→Image→*  *Morphological operations→Bottom-hat filtering* | Mode: 2D, full dataset  Strel element, shape: rectangle  Strel element, Size: 5 | 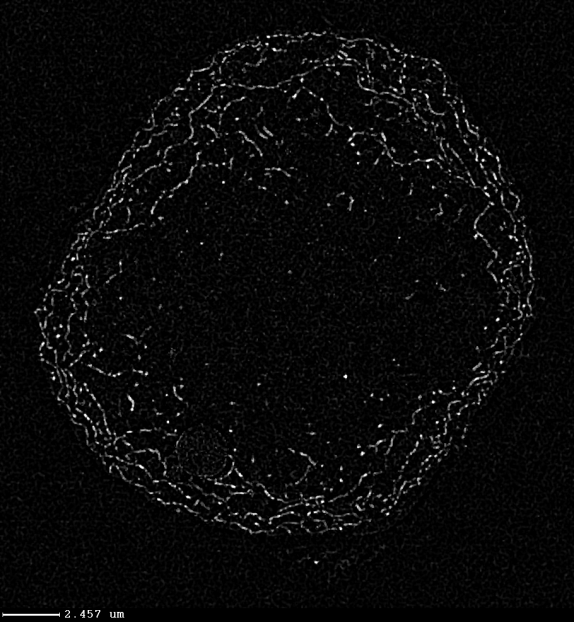 |

**Download link to the dataset:** [**http://mib.helsinki.fi/tutorials/mitoticcell_segmentation/MitoticCell_Segmentation_workflow.zip**](http://mib.helsinki.fi/tutorials/mitoticcell_segmentation/MitoticCell_Segmentation_workflow.zip)

| 3a. | **Hessian based Frangi Vesselness filter in the XY plane**  to segment ER tubules and sheet remnants that are perpendicular to the XY plane  *Where in MIB:*  *Mask Generators Panel (turned on using a popup menu in the Directory Contents panel)→Frangi filter→Do It* | Mode: 2D all  Range: 1-2  Ratio: 1  beta 1: .55  beta 2: 13  B/W thresholding: 0.15  Object size limit: 12  Black on while: unchecked | 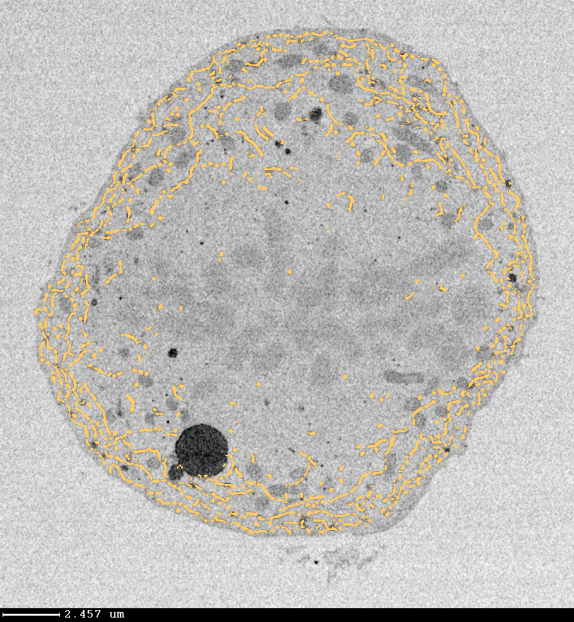  The segmented ER plotted against the original dataset |
| --- | --- | --- | --- |
| 3b. | **Hessian based Frangi Vesselness filter in the ZX plane**  to segment ER tubules and sheet remnants that are perpendicular to the ZX plane  *Where in MIB:*  ***a)*** *Toolbar→ZX button to change orientation*  ***b)*** *Mask Generators Panel→Frangi filter→the right mouse click above the Do It button and select “Generate a new mask and add it to the existing mask”*  *Assign the generated mask to the ER material of the model* | Range: 1-2  Ratio: 1  beta 1: .55  beta 2: 13  B/W thresholding: 0.15  Object size limit: 12  Black on while: unchecked |  |
| 3c. | **Add a new material “ER” to the model and assign results of the Frangi filter**  *Where in MIB:*  ***a)*** *Segmentation panel→”+”→”ER” →OK*  ***b)*** *Press the Shift+A shortcut to add selection to ER* | Segmentation Panel*→*  *Add to: 1* |  |
| 3d. | **Filter the results and save the model**  ***a)*** *Segmentation Panel→ER→*  *Right mouse click→Get Statistics…*  ***b)*** *Save model to the hard drive, Menu→Models→Save model as…* | Slices: Whole volume  Detect Objects: 3D objects  Press the Run button  - Highlight Range: 1 – 200  - Press the Do button to highlight the objects  - Click on the main window of MIB and press Shift+S to subtract highlighted objects from the ER material | 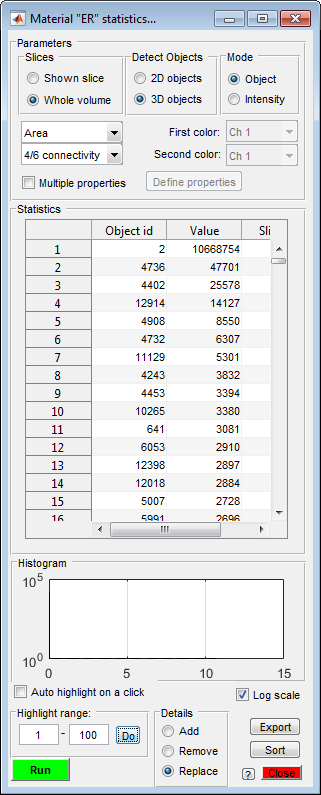 |
| 4. | **Subtract the model of ER from the anisotropically filtered dataset**  to simplify the data  *Where in MIB:*  ***a)*** *Load anisotropically filtered dataset, stored to the hard drive in step 1.*  ***b)*** *Load the model stored in the step 3b Menu→Models→Load model*  ***c)*** *Select the “ER” material and replace with the background the corresponding areas in the dataset.* | Segmentation Panel→Select from→1→right mouse click→NEW selection (ALL)  *Menu→Selection→Replace selected area in the image*  New intensity: 190  Slice number: 0  Color channels: 1 | 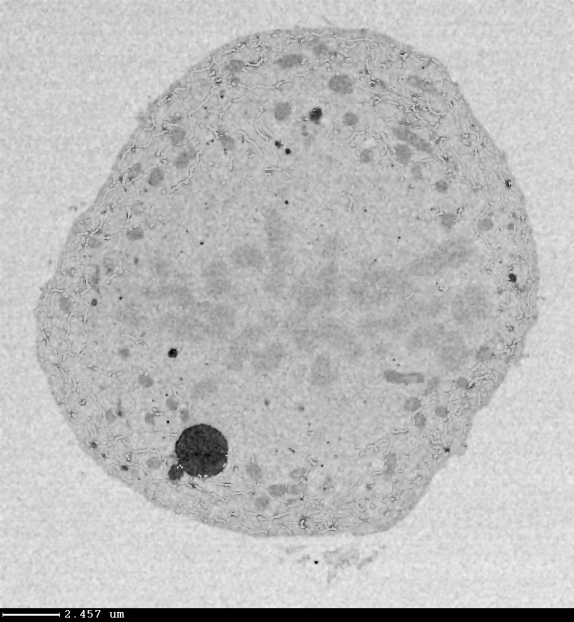 |
| 5. | **Black and white thresholding**  to segment dark, heavily stained organelles, such as lipid droplets and lysosomes  *Where in MIB:*  *Segmentation Panel→Selection type→BW Theresholding* | Low Lim: 0  High Lim: 131  all: checked  Select from: Ext  Fix selection to material: checked  *Remember uncheck the Fix selection to material checkbox after thresholding* | 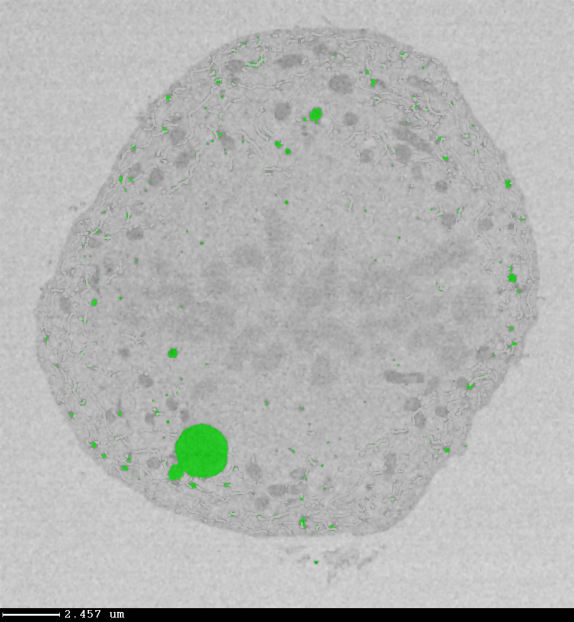 |
| 6. | **Erode and dilate in 3D**  to remove small irrelevant objects  *Where in MIB:*  ***a)*** *Selection Panel→Er*  ***b)*** *Selection Panel→Di*  ***c)*** *Assign results to the new material (LD) of the model* | Color channel: Ch 1  3D: checked  Strel: 3;2  *similar to 3c, but*  Segmentation Panel*→*  *Add to: 2* | 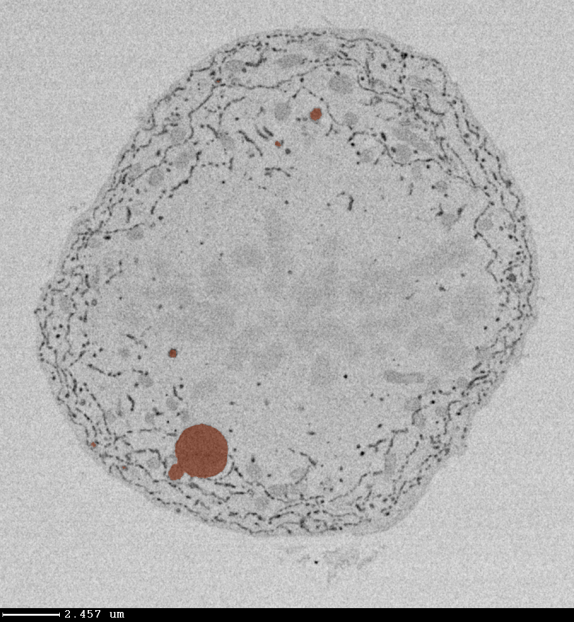  The segmented lipid droplets and lysosomes plotted against the original dataset |

| 7. | **Subtract the model of LD from the dataset**  to further simplify the data  *Where in MIB:*  ***a)*** *Select the “LD” material and replace with the background the corresponding areas in the dataset.* | Segmentation Panel→Select from→2→right mouse click→NEW selection (ALL)  *Menu→Selection→Replace selected area in the image*  New intensity: 190  Slice number: 0  Color channels: 1 | 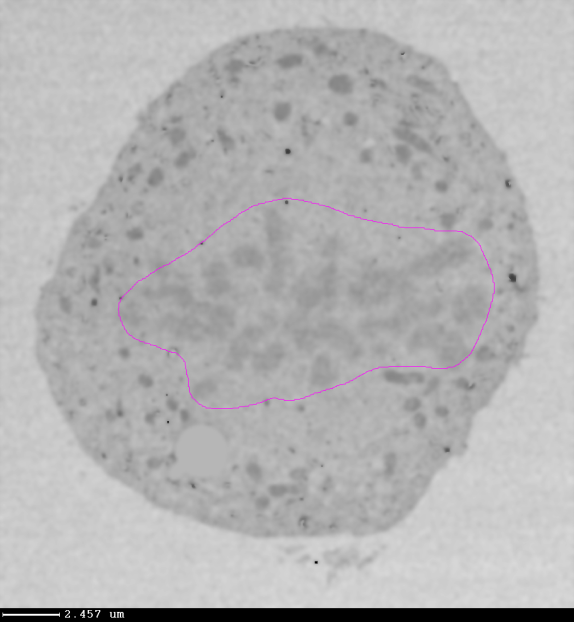  The masked area is indicated with magenta contour |
| --- | --- | --- | --- |
| 8a. | **Perona-Malik**  **anisotropic diffusion filtering**  additional filtering  *Where in MIB:*  ***a)*** *Image Filters Panel→Perona Malik anisotropic diffusion→Filter* | Type: Regions  Number of iterations (Iter): 20  Edge stopping parameter (K): 6  Diffusion step (lambda): 0.25  All: checked |  |
| 8b. | **The Brush tool and the shape interpolation**  to mask the central part of the cell containing chromosomes  *Where in MIB:*  *a) Segmentation Panel→Selection type→Brush*  *b) Menu→Selection→*  *Interpolate as Shapes* | NA |  |
| 8c. | **Local Black and White thresholding within the masked area**  to segment chromosomes  *Where in MIB:*  *Segmentation Panel→Selection type→BW Theresholding* | Low Lim: 0  High Lim: 173  all: checked  Masked area: checked | 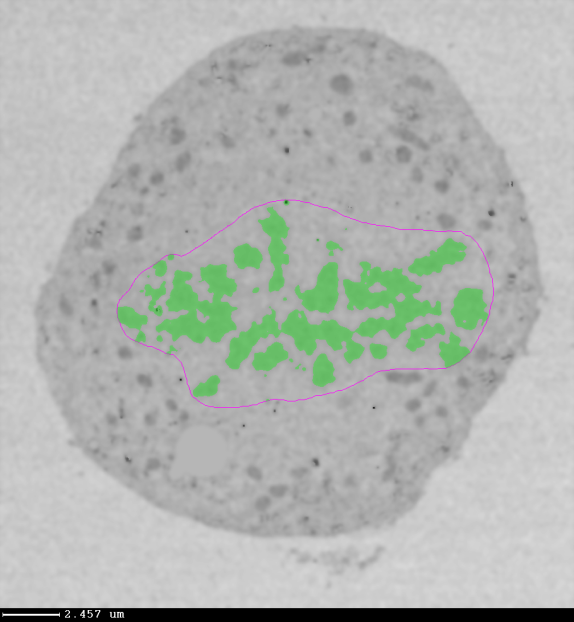 |

| 9a. | **Erode and dilate in 3D**  to remove small irrelevant objects  *Where in MIB:*  ***a)*** *Selection Panel→Er*  ***b)*** *Selection Panel→Di*  ***c)*** *Assign results to the new material (Chromosomes) of the model* | Color channel: Ch 1  3D: checked  Strel: 4;2  *similar to 3c, but*  Segmentation Panel*→*  *Add to: 3* | 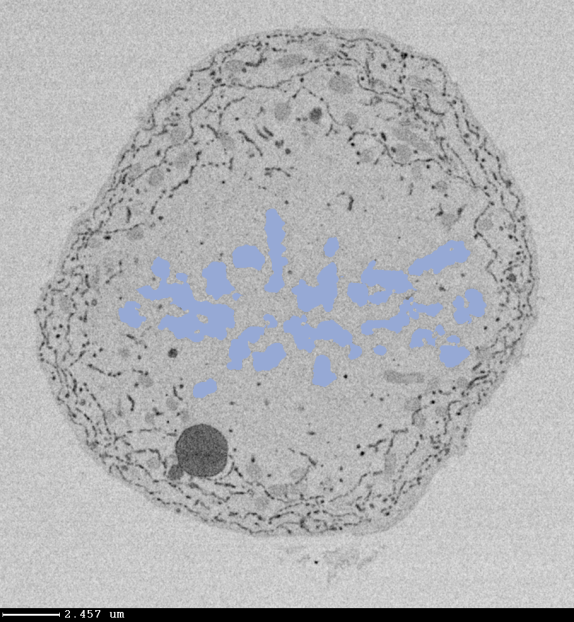  The segmented chromosomes plotted against the original dataset |
| --- | --- | --- | --- |
| 9b. | **Filtering results based on size and intensity properties of the segmented objects**  to eliminate too small or too dark objects  *Where in MIB:*  *Segmentation Panel→Chromosomes→*  *Right mouse click→Get Statistics…* | Slices: Whole volume  **a)** Detect objects: 2D objects  Mode: Intensity  Parameter: MeanIntensity  Remove objects darker than 165  **b)** Detect objects: 2D objects  Mode: Object  Parameter: Area  Remove objects smaller than 150  **c)** Detect objects: 3D objects  Mode: Object  Parameter: Area  Remove all small objects |  |
| 10. | **Subtract the model of Chromosomes from the dataset**  to further simplify the data  *Where in MIB:*  ***a)*** *Select the “Chromosomes” material and replace with the background the corresponding areas in the dataset.* | Segmentation Panel→Select from→3→right mouse click→NEW selection (ALL)  *Menu→Selection→Replace selected area in the image*  New intensity: 179  Slice number: 0  Color channels: 1 | 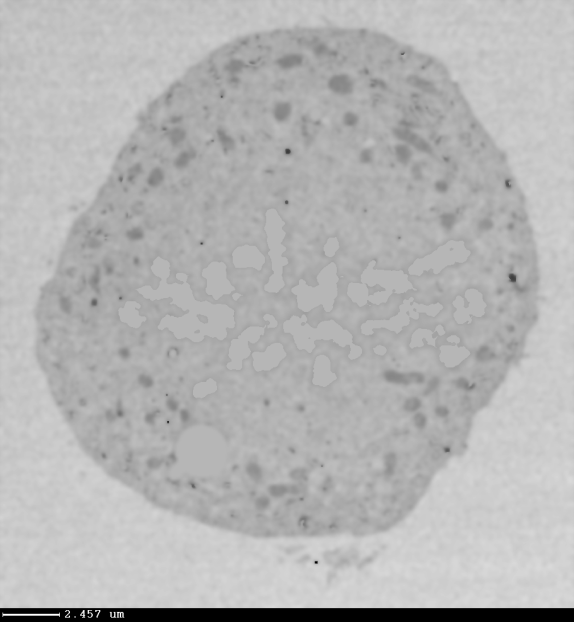 |
| 11a. | **Perona-Malik**  **anisotropic diffusion filtering**  additional filtering  *Where in MIB:*  ***a)*** *Image Filters Panel→Perona Malik anisotropic diffusion→Filter* | Type: Regions  Number of iterations (Iter): 20  Edge stopping parameter (K): 15  Diffusion step (lambda): 0.25 | 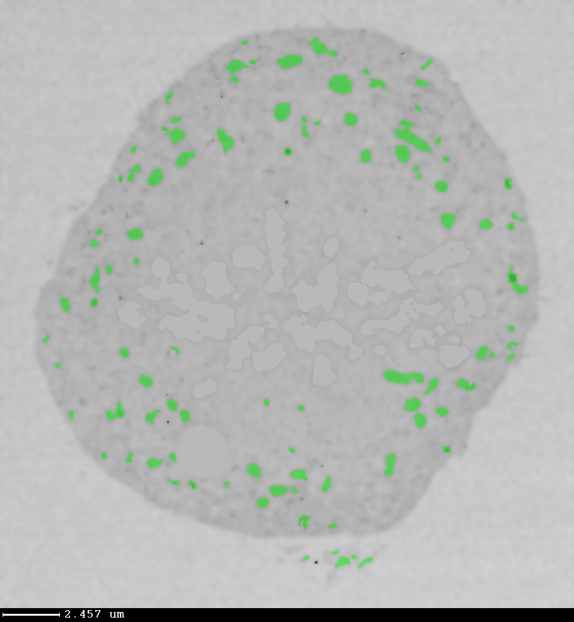 |
| 11b. | **Strel mask generator**  to segment mitochondria  (*the* *slowest step*)  *Where in MIB:*  *Mask Generators Panel→Strel filter* | Strel: 25  B/W threshold: 0.07  Size limit: 50  Mode: 3D  Black on white: checked  Press the “Do it” button |  |
| 12a. | **Select Mask that does not belong to any other material and assign to a new material of the model**  *Where in MIB:*  ***a)*** *Highlight “Ext” in the Segmentation Panel using the left mouse button*  ***b)*** *Menu→Mask→to Selection…→All Frames→Replace*  ***c)*** *Add a new material: “Mitochondria”*  ***d)*** *Press the Shift+A shortcut to assign selection to the new material* | Segmentation Panel→Fix selection to material: checked  Segmentation Panel→Fix selection to material: unchecked | 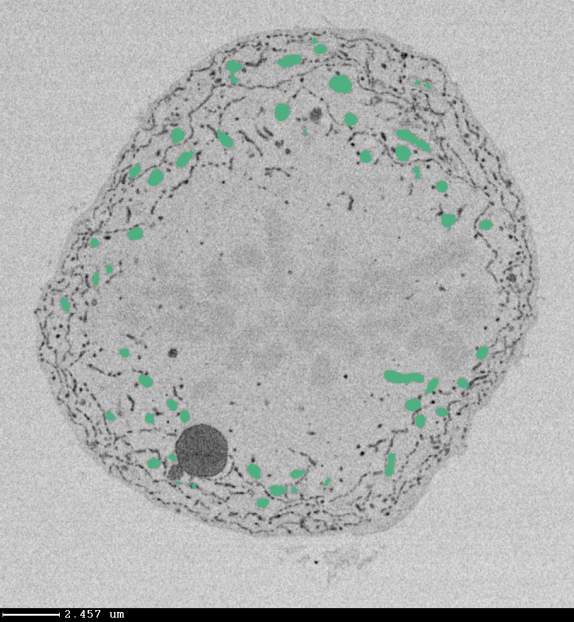  The segmented mitochondria plotted against the original dataset |
| 12b. | **Filtering results based on size properties of the segmented objects**  to eliminate too small objects  *Where in MIB:*  *Segmentation Panel→Mitochondria→*  *Right mouse click→Get Statistics…* | Slices: Whole volume  Mode: Object  Parameter: Area  **a)** Detect objects: 2D objects  Remove objects smaller than 50 pixels  **b)** Detect objects: 3D objects  Remove objects smaller than 1200 pixels |  |
| 12c. | **Erode and dilate in 3D**  to remove small irrelevant objects  *Where in MIB:*  ***a)*** *Select the “Mitochondia” material*  ***a)*** *Selection Panel→Er x2 times*  ***b)*** *Selection Panel→Di x2 times* | Segmentation Panel→Select from→4→right mouse click→NEW selection (ALL)  *Selection Panel:*  Color channel: Ch 1  3D: checked  Strel: 1;1 |  |
| 12d. | **Smooth selection**  *Where in MIB:*  *Menu→Selection→Smooth selection* | Mode: 3d  XY Kernel size: 5  Z Kernel size: 5  Sigma: 5 |  |
| 12e. | **Replace the “Mitochondria” material with the current selection**  ***a)*** *Segmentation Panel→Add to: 4*  ***b)*** *Press the Shift+R shortcut to replace the material* |  |  |
| 12f. | **Filtering results based on size properties of the segmented objects**  to eliminate too small objects  *Due to the low contrast of mitochondria, an additional manual poling using the Brush tool is required after this step* | As in step 12b. |  |
| 13. | **Visualization of the model**  *Where in MIB:*  *Menu→Models→Save model as…* | Format: AmiraMesh binary |  |
