# Supplementary material for: Microscopy Image Browser: A Platform for Segmentation and Analysis of Multidimensional Datasets
Source: PLoS Biol. 2016 Jan 4;14(1):e1002340. doi: 10.1371/journal.pbio.1002340 (PMC4699692; doi:10.1371/journal.pbio.1002340)
Supplement: S2 Table — (DOCX) [file pbio.1002340.s003.docx]

| **Name of the package** | **Authors and link** | **License,**  **year** |
| --- | --- | --- |
| Accurate Fast Marching | Dirk-Jan Kroon, University of Twente  <http://www.mathworks.se/matlabcentral/fileexchange/24531-accurate-fast-marching> | FreeBSD,  2011 |
| Anisodiff | Peter Kovesi, Centre for Exploration Targeting, School of Earth and Environment, The University of Western Australia  <http://www.csse.uwa.edu.au/~pk/Research/MatlabFns/#anisodiff> | MIT,  2000-2002 |
| Bio-Formats | Melissa Linkert, Curtis Rueden et al.  <http://loci.wisc.edu/software/bio-formats> | GPL,  2002-2015 |
| bwdistsc | Yuriy Mishchenko, Toros University  <http://www.mathworks.se/matlabcentral/fileexchange/15455-3d-euclidean-distance-transform-for-variable-data-aspect-ratio> | FreeBSD,  2007-2013 |
| DrawRegionBoundaries | Peter Kovesi, Centre for Exploration Targeting, School of Earth and Environment, The University of Western Australia  <http://www.peterkovesi.com/projects/segmentation/> | MIT,  2013 |
| Export_fig | Oliver Woodford and Yair Altman | ModifiedBSD  2014-2015 |
| Extrema, extrema2 | Carlos Adrian Vargas Aguilera, Universidad de Guadalajara  <http://www.mathworks.com/matlabcentral/fileexchange/12275-extrema-m-extrema2-m> | ND,  2006-2007 |
| Fast 3D/2D Region Growing (MEX) | Christian Wuerslin, Stanford University  <http://www.mathworks.com/matlabcentral/fileexchange/41666-fast-3d-2d-region-growing--mex-> | ModifiedBSD,  2013-2015 |
| Fast/Robust Template Matching | Dirk-Jan Kroon, University of Twente  <http://www.mathworks.com/matlabcentral/fileexchange/24925-fastrobust-template-matching> | FreeBSD,  2009-2011 |
| FindJObj | Yair Altman  <http://www.mathworks.com/matlabcentral/fileexchange/14317-findjobj-find-java-handles-of-matlab-graphic-objects> | FreeBSD,  2007-2013 |
| Hardware accelerated 3D viewer for MATLAB | Jean-Yves Tinevez, Institut Pasteur  <http://www.mathworks.com/matlabcentral/fileexchange/32344-hardware-accelerated-3d-viewer-for-matlab> | FreeBSD,  2011 |
| Hessian based Frangi Vesselness filter | Marc Schrijver and Dirk-Jan Kroon, University of Twente  <http://www.mathworks.com/matlabcentral/fileexchange/24409-hessian-based-frangi-vesselness-filter> | FreeBSD,  2001-2009 |
| IceImarisConnector | Aaron C. Ponti, ETH Zurich  <http://www.scs2.net/next/index.php?id=110> | GPL,  2013 |
| Image Edge Enhancing Coherence Filter | Dirk-Jan Kroon, Pascal Getreuer University of Twente  <http://www.mathworks.com/matlabcentral/fileexchange/25449-image-edge-enhancing-coherence-filter-toolbox> | FreeBSD,  2009 |
| Image Measurement Utility | Jan Neggers, Eindhoven Univeristy of Technology  <http://www.mathworks.com/matlabcentral/fileexchange/25964-image-measurement-utility> | FreeBSD,  2009-2014 |
| Imageviewer | Jiro Doke, Mathworks  <http://www.mathworks.com/matlabcentral/fileexchange/13000-imageviewer> | ModifiedBSD,  2010 |
| Imclipboard | Jiro Doke, MathWorks  <http://www.mathworks.com/matlabcentral/fileexchange/28708-imclipboard> | ModifiedBSD,  2010 |
| Imgaussian | Dirk-Jan Kroon, University of Twente  <http://www.mathworks.com/matlabcentral/fileexchange/25397-imgaussian> | FreeBSD,  2009 |
| Local normalization | Guanglei Xiong at Tsinghua University, Beijing  <http://www.mathworks.com/matlabcentral/fileexchange/8303-local-normalization> | ND,  2005 |
| MatTomo, a part of PEET | Boulder Laboratory for 3-D Electron Microscopy of Cells  <http://bio3d.colorado.edu/PEET/index.html> | GPL,  2006-2010 |
| MAXFLOW/MINCUT algorithm, v2.22 | Yuri Boykov, University of Western Ontario and Vladimir Kolmogorov, Microsoft research, Cambridge  <http://pub.ist.ac.at/~vnk/software.html> | GPL,  2001-2014 |
| MAXFLOW/MINCUT Matlab wrapper | Michael Rubinstein, Google | FreeBSD, 2008 |
| Membrane detection with Random Forest | Verena Kaynig, Harvard School of Engineering and Applied Sciences  <http://www.kaynig.de/demos.html> | GPL,  2010 |
| MTOC++ | Martin Drohmann (Universität Münster), Daniel Wirtz (Universität Stuttgart)  <http://www.mathworks.com/matlabcentral/fileexchange/33826-mtoc++-doxygen-filter-for-matlab-and-tools> | FreeBSD,  2011-2013 |
| NRRD Format File Reader | Jeff Mather  <http://www.mathworks.com/matlabcentral/fileexchange/34653-nrrd-format-file-reader> | ModifiedBSD,  2012 |
| Projects:MATLABSlicerExampleModule, to read NRRD format | John Melonakos  <http://www.na-mic.org/Wiki/index.php/Projects:MATLABSlicerExampleModule> | GPL,  2012 |
| Random Forest Matlab | Abhishek Jaiantilal  <https://code.google.com/p/randomforest-matlab/> | GPL,  2012 |
| Region Adjacency Graph (RAG) | David Legland, INRA, France  <http://www.mathworks.com/matlabcentral/fileexchange/16938-region-adjacency-graph--rag-> | FreeBSD,  2007-2013 |
| regionprops3 | Chaoyuan Yeh, University of Southern California  <http://www.mathworks.com/matlabcentral/fileexchange/47578-regionprops3> | FreeBSD,  2014 |
| Render RGB text over RGB or Grayscale Image | Davide Di Gloria, Universita di Genova  <http://www.mathworks.com/matlabcentral/fileexchange/26940-render-rgb-text-over-rgb-or-grayscale-image> | FreeBSD,  2010 |
| SLIC, superpixels | Radhakrishna Achanta, Appu Shaji, Kevin Smith, Aurelien Lucchi, Pascal Fua, and Sabine Süsstrunk, Ecole Polytechnique Federale de Lausanne (EPFL), Switzerland  <http://ivrl.epfl.ch/supplementary_material/RK_SLICSuperpixels/index.html> | ModifiedBSD,  2015 |
| View3D | Torsten Vogel  <http://www.mathworks.com/matlabcentral/fileexchange/334-view3d-m> | FreeBSD,  1999 |
| XLSWRITE Improved | Barry Dillon, AON Insurance Brokers  <http://www.mathworks.com/matlabcentral/fileexchange/27236-improved-xlswrite-m> | FreeBSD,  2010 |

**Abbreviations:**

**FreeBSD**, Berkeley Software Distribution, 2-clause license, GPL compatible

**GPL**, GNU General Public License, version 2

**ModifiedBSD**, Berkeley Software Distribution, 3-clause license, GPL compatible

**MIT**, a free software license originating at the Massachusetts Institute of Technology, GPL compatible

**ND**, no data
